# Molecular Dynamics Simulation of Texture Contact Friction Between Crystalline Silicon Layers for Application in Micro-Nano System Devices

**DOI:** 10.3390/molecules31010091

**Published:** 2025-12-25

**Authors:** Jinping Zhang, Minghui Tan, Shan Yuan, Fei Wang, Yu Jia, Xiaolei Wang

**Affiliations:** 1 Henan Provincial Key-Laboratory of Nano-Composite and Applications, Institute of Nanostructured Functional Materials, Faculty of Engineering, Huanghe Science and Technology College, Zhengzhou 450006, China; mht17634517502@126.com (M.T.); ys620s@126.com (S.Y.); wangf157@hhstu.edu.cn (F.W.); 2 School of Materials Science and Engineering, Henan University of Science and Technology, Luoyang 471023, China; 3 Institute of Quantum Materials and Physics, Henan Academy of Science, Zhengzhou 450046, China; 4 Key Laboratory for Special Functional Materials of Ministry of Education, School of Physics and Electronics, Henan University, Kaifeng 475001, China; 5 Henan Zhongfu Industrial Co., Ltd., Gongyi 452100, China; wangxiaolei@zfsy.com.cn

**Keywords:** molecular dynamics, reactive force field, crystalline silicon, friction, sinusoidal surface

## Abstract

Silicon is commonly used in micro/nano-electromechanical system (MEMS/NEMS) devices. Because detailed information about the friction interface in these systems is lacking, the relationship between texture shape and friction remains unclear. In this study, molecular dynamics simulations were performed to investigate the dry-friction tribological behavior of crystalline silicon, focusing on the effects of surface roughness, normal load, and sliding speed. The results show that between normal loads of 4 GPa and 8 GPa, the average frictional force exhibits significant nonlinear behavior under a sliding speed of 0.2 Å/ps. The approximate steady value of the friction coefficient is 0.39, which is in good agreement with the experimental result of 0.37. Under a normal load of 5 GPa, the friction force increases linearly from 110 nN at 0.05 Å/ps to 311 nN at 2 Å/ps. In addition, in systems with sinusoidal surface roughness, the amplitude has a greater effect on the frictional properties than the period. Among the four rough surfaces studied, A10T32 exhibits the lowest friction force and friction coefficient. This provides theoretical support for the further design of MEMS/NEMS devices with long operational lifetimes.

## 1. Introduction

Friction is a process of energy dissipation from contact interfaces during relative motion. It exists widely in nature and engineering applications [1,2]. Understanding and controlling tribological behavior is essential for improving efficiency and durability in various systems, ranging from industrial machinery to micromechanical devices [3,4,5]. Silicon is widely used in the core structural materials of micro/nano-electromechanical system (MEMS/NEMS) devices [6,7,8,9] due to its excellent semiconductor characteristics and good mechanical properties. However, silicon exhibits poor tribological behavior due to its high coefficient of friction (COF) and susceptibility to wear. This limits its application in MEMS/NEMS devices. Hence, mitigation of the COF and wear of silicon is crucial for improving the overall performance, reliability, and operational lifetime of MEMS/NEMS devices.

Many researchers now use lubricants [10,11,12,13,14,15,16] or solid lubricating coatings [17,18] to avoid the high COF of silicon and silica. For instance, vapor-phase lubrication with alcohols has been proved to significantly reduce friction and wear on silica surfaces in NEMS devices [10,11,12]. Saramago et al. [13,14,15,16] demonstrated that ionic liquids, as additives in base oil, effectively reduce the COF of silicon surfaces and improve the lubricity of silicon surfaces. Solid lubricating coatings, especially those based on transition metal disulfides such as molybdenum disulfide (MoS2) [17,18], can reduce friction and wear under harsh operating conditions where traditional lubricants may fail. In addition, the COF of silicon and silica can be reduced by adjusting the surface contact area.

With the advent of atomic force microscopy (AFM), the size of NEMS devices has been reduced to the nanoscale, where the ratio of surface area to volume increases sharply. This means that surface effects (such as adhesion, friction, and wear) play an important role in determining the tribological properties of silicon and silica. The existing investigations have demonstrated that surface roughness has a significant impact on frictional forces [19,20,21]. Surface texturing, which effectively reduces the real contact area between interacting surfaces, is a highly efficient method for decreasing frictional forces at the nanoscale [22,23,24]. Winkless et al. [25] performed the microtribology sliding experiments on various micropatterned silicon surfaces. It was observed that the high friction values seen in many experiments were linked to substantial damage to the structure of the patterned silicon surface. Yoon et al. [26] investigated the micro/nano-frictional properties of Si(100) surfaces that had been topographically modified. The findings indicated that for nano-patterned surfaces, the decrease in friction was partly due to the physical (geometrical) reduction in the contact area. They also fabricated the silicon micro-patterns on Si(100) wafers using photolithography and deep reactive ion etching fabrication techniques [27]. The micro-friction behaviors of the surfaces were evaluated, and the results indicate that a combination of both the topographical and chemical modification is very effective in reducing the friction. Although these experimental investigations can determine how the textured surfaces affect the frictional property, they cannot clearly illustrate the lattice changes in materials, the formation and breaking of chemical bonds, and other friction behavior at the atomic level.

In this study, the tribological behavior between the planar upper Si film and lower Si films with different roughness values under dry friction conditions, at different normal loads and sliding speeds are investigated using molecular dynamics simulations. A frictional model is established, and the friction process is simulated. The effects of roughness, normal load, and sliding speed on friction properties are studied, and the relationships between average friction force and friction coefficient and normal load and sliding speed are obtained. The rough surface with the best friction performance is determined. This provides theoretical guidance for designing Si film patterns with optimal friction performance, effectively improving the operational lifetime of MEMS/NEMS.

## 2. Results and Discussion

### 2.1. Effects of Applied Normal Load

**Figure 1:**
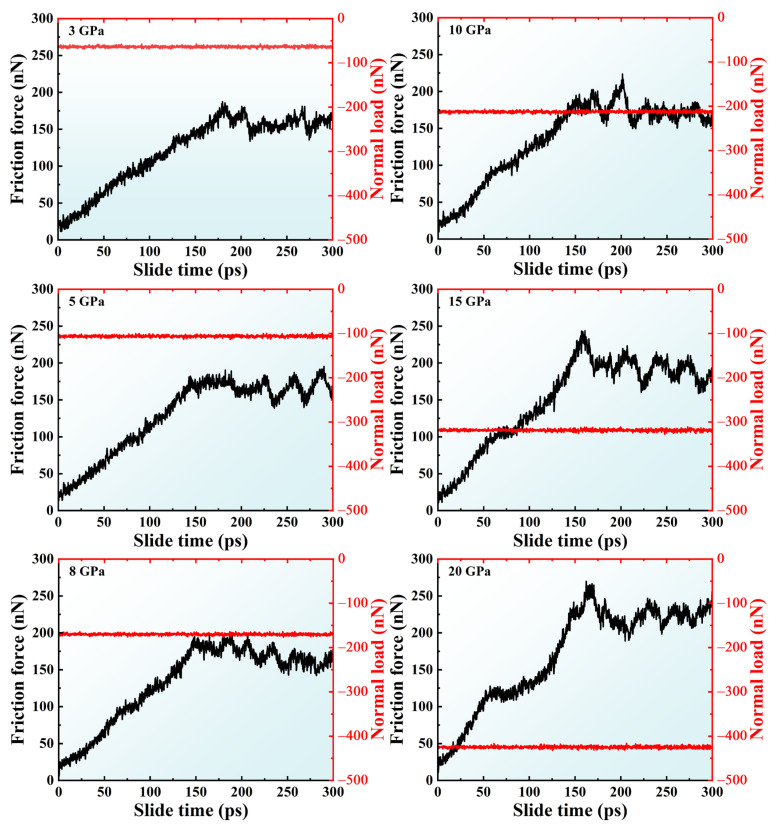
Friction force and normal load curves of the A5T32 system with sliding time under different applied pressures.

**Figure 2:**
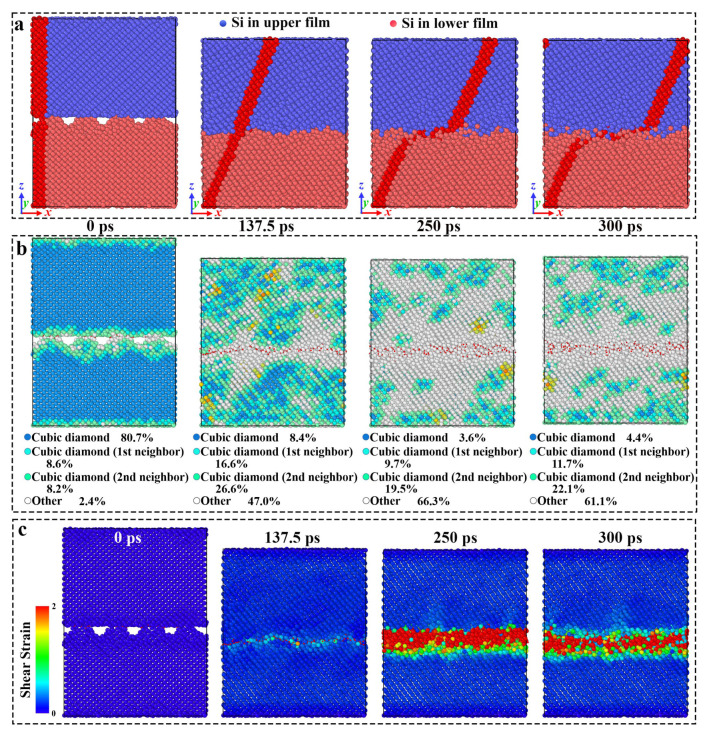
The (**a**) snapshots of atomic configuration, (**b**) the lattice structure, and (**c**) the shear strain evolution of the A5T32 system during the friction process at a normal load of 5 GPa.

In order to investigate the effects of pressure on the tribological behavior of crystalline silicon under dry friction conditions, the friction curves for the A5T32 system at a sliding speed of 0.2 Å/ps under various contact pressures are shown in Figure 1. Friction force, *F_f_*, is obtained by summing the tangential forces of all atoms in the upper fixed layer along the sliding direction; and normal load force, *F_n_*, is obtained by summing the normal forces of all the atoms in the bottom fixed layer along the z direction during the friction process [28]. A negative value is used to represent the normal load force as its direction is opposite to that of the positive z-axis. From Figure 1, it can be seen that when the contact pressure is below 10 GPa, the *F_f_* rises significantly at the beginning of the sliding process. This is due to Si–Si bond stretching leading to the Si distortion, which increases the sliding resistance. This can be seen directly in Figure 2. Figure 2a shows snapshots of the atomic configuration evolution of the A5T32 system during friction at a normal load of 5 GPa. The red part is the Si atom labeling performed before the relative sliding occurs, which is used to represent the deformation of the Si film. It can be seen that the red mark is in the tilted state at 137.5 ps, indicating that the contact interface of the Si film does not slide and is in the shear deformation state, resulting in increased sliding resistance and increased friction force *F_f_*. When the friction force reaches the stability value, the friction force curve starts to fluctuate. This is because the contact interface disappears, and the upper and lower Si films are completely mixed, forming a merged structure that maintains the friction force, as shown in the 250 ps and 300 ps snapshots in Figure 2. This is consistent with the findings reported in References [29,30]. Figure 2b illustrates the alteration in the lattice structure of the A5T32 system during the friction process. The red stick represents the bonds formed between the upper- and lower-interface silicon atoms. It highlights that before the sliding (at 0 ps), the lattice structure of the Si friction films predominantly comprises a cubic diamond lattice, constituting approximately 80.7% of the structure, while the proportion of interface atoms exhibiting an amorphous phase is approximately 2.4%. However, after 137.5 ps of friction at a normal load of 5 GPa, the cubic diamond structure diminishes to 8.4%, and there is an increase in the contribution of amorphous atoms up to approximately 47.0%. Furthermore, the presence of the cubic diamond (first neighbor) and (second neighbor) lattice, accounting for 16.6% and 26.6%, respectively, indicate that the lattice distortion occurred locally. In addition, the number of atomic bonds increases from 43 to 316, which results in a continuous increase in frictional force, consistent with the phenomenon observed in Figure 1. At 250 ps and 300 ps, the cubic diamond atoms account for 3.6% and 4.4%, respectively. The amorphous structure accounted for 66.3% and 61.1%, respectively. The results show that the structure ratio has reached dynamic stability, and the irreversible microstructural evolution toward distortion and amorphousness underlies the increased and stabilized friction force. The friction mechanism at the interface has changed from crystal plasticity to amorphous viscous flow. The shear strain distribution of the A5T32 system at different friction times is shown in Figure 2c. As the sliding time increases, the shear strain is concentrated in the interface region. At 137.5 ps, small shear strain occurs in the interface region. As shown in Figure 2a,b, even at a low shear strain, the interface undergoes plastic deformation, and the resulting energy dissipation is primarily responsible for the rise in macroscopic friction. With increasing sliding time, the interfacial shear strain evolves into a stronger state, characterized by a relatively parallel and uniform distribution. Notably, the higher shear strain observed at 250 ps corresponds to a higher friction force (167.9 nN), while the lower strain at 300 ps corresponds to a lower force (152.6 nN). These findings demonstrate that under a 5 GPa normal load, the proportion of cubic diamond (first/second neighbor) atoms rises during friction, accompanied by the onset of plastic deformation, which increases the friction force. Concurrently, the amorphous phase at the interface expands markedly and approaches dynamic stability, while shear strain increases and becomes more uniformly distributed, enabling the friction force to attain a dynamically steady state. This provides direct evidence that the steady-state friction behavior is governed by the steady-state microstructure.

**Figure 3:**
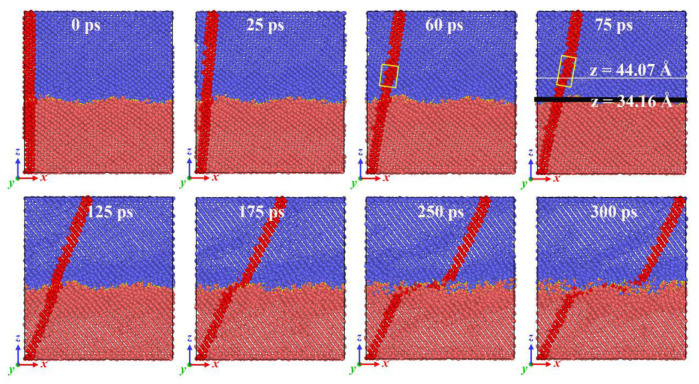
Snapshots of the atomic configuration evolution of the A5T32 system during the friction process at a normal load of 20 GPa. The red part is the Si atom marked before the relative sliding, which is used to demonstrate the deformation of the Si film, the blue represents the Si atoms of upper films, and the red is the Si atoms of lower films.

It is worth noting that when the contact pressure is higher than 10 GPa, the friction force curve undergoes obvious changes. At the beginning of sliding, a plateau appears as the friction increases; the greater the contact pressure, the longer the stability. To explain this phenomenon, Figure 3 shows snapshots of the atomic configuration evolution of the A5T32 system during the friction process at a normal load of 20 GPa. The yellow stick represents the bonds formed between the upper- and lower-interface silicon atoms. It can be seen that the red mark is in a tilted state at 25 ps, indicating that the contact interface of the Si film is in a state of shear deformation, which leads to an increase in sliding resistance, so the friction force, *F_f_*, increases. It was noted that at a contact pressure of 20 GPa in Figure 1, *F_f_* remains almost stable between 60 and 100 ps. This is because the shear deformation occurs in the Newtonian layer of the upper Si film (c and d snapshots of Figure 3). This region is located approximately 10 Å above the interface at a height of z ≈ 44.07 Å, which affects the friction force at the interface and keeps it unchanged. Then, the *F_f_* rises sharply, with a slight reduction in stability. An intuitive explanation can be given in Figure 3. The deformation of the thermostatic layer disappears as seen from the snapshot at 125 ps. Starting at 175 ps, the upper and lower Si films layers move relative to each other, and a merged structure forms, keeping the final friction force stable.

**Figure 4:**
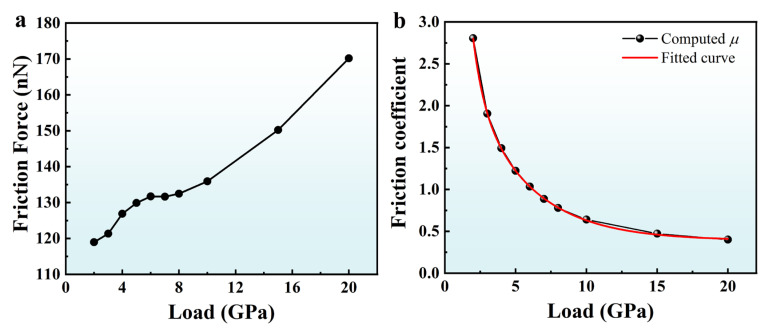
(**a**) Average friction force and (**b**) friction coefficient as a function of load pressure for a sliding speed for 0.2 Å/ps for the A5T32 system.

**Figure 5:**
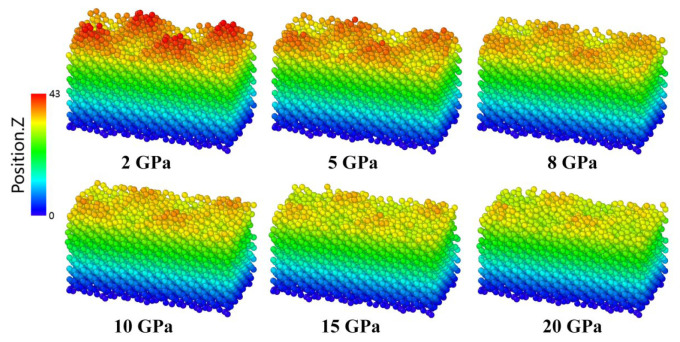
Snapshots of the atomic configuration in the lower contact surface of the A5T32 system after 10 ps under different normal loads (2, 5, 8, 10, 15, 20 GPa).

To compare the tribological properties of the A5T32 system with the different pressures, the average friction force, *F_f_*, and normal load, *F_n_*, are obtained using the average values of the friction curves in Figure 1. The average friction coefficient, *µ*, is calculated using the following equation: $\mu =\frac{F_{f}}{F_{n}}$ [28]. The computed *F_f_* and *µ* under different pressures for A5T32 system are shown in Figure 4. Figure 4a shows that the average friction force increases with the increase in the normal load, but it is not a completely linear relationship. This conclusion is consistent with the experimental results obtained by Kalin and Jan [31]. A clear deviation from linear behavior is observed in the average friction force, beginning at a normal load of 4 GPa. However, the response shows a return to a quasi-linear regime as the normal load approaches 8 GPa. From Figure 4b, the computed results show that the average friction coefficient of the A5T32 system decreases with the contact pressure, consistent with the conclusion of Li et al. [32] This can be explained by the smoothness of the contact surface under different contact pressures, as shown in Figure 5. Figure 5 provides a snapshots of the atomic configuration in the lower contact surface of the A5T32 system under different contact pressures. It is clear from the figure that at a low contact pressure (2 GPa), the contact interface is characterized by sharp asperities, resulting in a limited real contact area and a high friction coefficient due to elevated local shear stresses. With increasing contact pressure, these asperities deform and flatten, which enlarges the real contact area and smoothens the interface. Especially at contact pressures of 15 GPa and 20 GPa, the smooth surface creates stronger covalent bonds (285 and 345, respectively) at the interface, causing transient shear deformation within 10 Å above the interface during friction (Figure 3). Consequently, the friction coefficient decreases because the growth of the real contact area cannot keep pace with the applied load. Through fitting, we obtained the relationship between the average friction coefficient and the contact pressure as follows:

(1) $$\mu =\mu_{0}+A_{1}e^{-\lambda_{1}N}+A_{2}e^{-\lambda_{2}N}$$

where, *µ* is the average friction coefficient, *N* is the contact pressure, *µ*_0_ is the approximate steady value of friction coefficient, $e^{-\lambda_{1}N}$ is the fast decay term, *λ*_1_ is the fast deceleration rate, $e^{-\lambda_{2}N}$ is the slow decay term, *λ*_2_ is the slow deceleration rate, and *A*_1_ and *A*_2_ are the amplitude coefficients of the fast-decaying and slow-decaying terms, respectively. The value of *µ*_0_, *A*_1_, *A*_2_, *λ*_1_, and *λ*_2_ are 0.39, 10.87, 2.80, 1.37 and 0.25, respectively, which are obtained from the fitting curve in Figure 4b. It is worth noting that the approximate steady values of friction coefficient is 0.39, which is in good agreement with the experimental values of 0.44 (pristine surface) and 0.37 (patterned surface) obtained in a vacuum environment in Reference [33].

### 2.2. Effects of Friction Velocity

**Figure 6:**
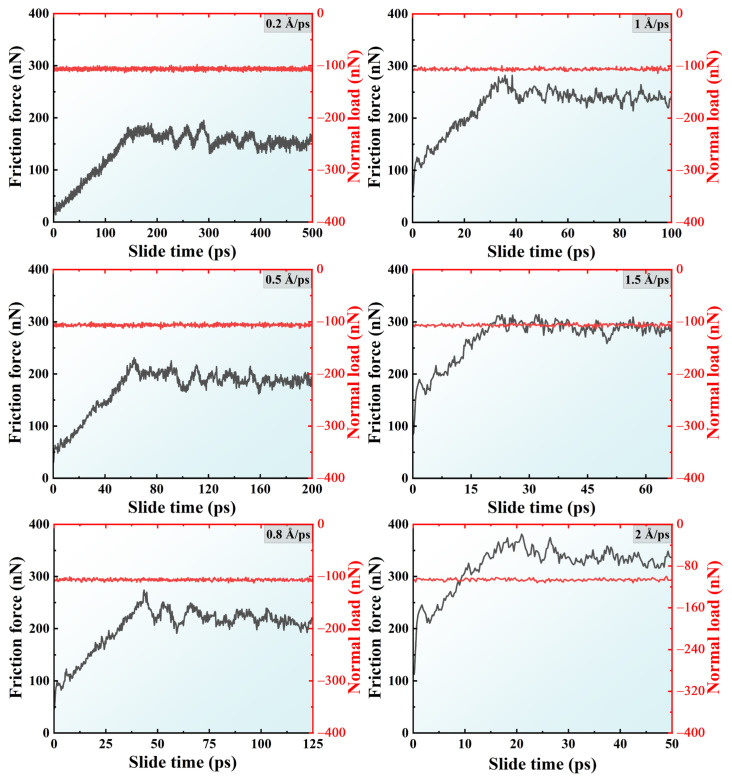
Friction force and normal load curves of the A5T27 system with sliding time under different sliding speeds.

**Figure 7:**
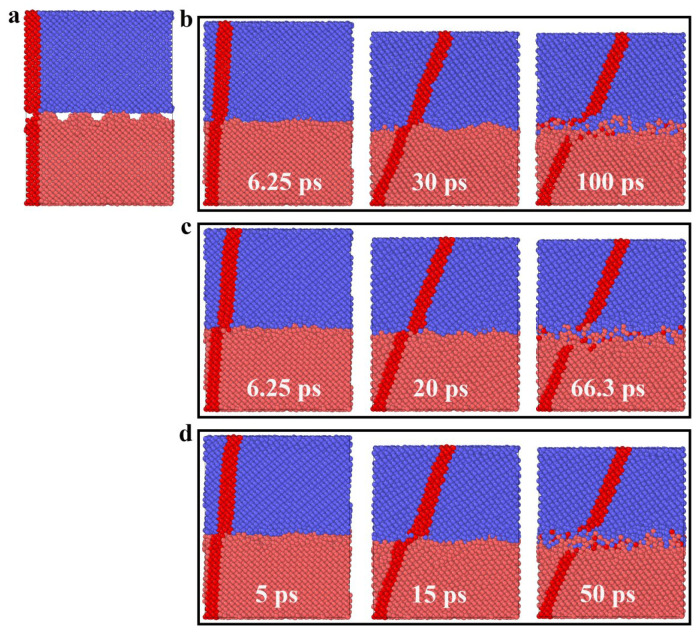
Snapshots of the atomic configuration evolution of the A5T32 system during the friction process at different sliding speeds: (**b**) 1 Åps^−1^, (**c**) 1.5 Åps^−1^, and (**d**) 2 Åps^−1^. (**a**) is the atomic configuration before the friction. The red part is the Si atom marked before the relative sliding, which is used to demonstrate the deformation of the Si film, the blue represents the Si atoms of upper films, and the red is the Si atoms of lower films.

In order to investigate the effects of sliding speed on the tribological behavior of crystalline silicon under dry friction conditions, the friction curves for the A5T32 system at various sliding speeds under 5 GPa are presented in Figure 6. As depicted in the Figure 6, the friction processes at different friction speeds can roughly be divided into two stages: the early friction increase stage and the friction stabilization stage. With the sliding speed increase, the early stage of friction can also be divided into two stages: the planar upper Si film completely touches the rough surface of the lower Si film (the contact area reaches the maximum); after that, the friction force rises steadily. This can be seen intuitively, as shown in Figure 7. Figure 7 provides snapshots of the atomic configuration evolution of the A5T32 system during the friction process at sliding speeds of 1, 1.5, and 2 Å/ps, respectively.

**Figure 8:**
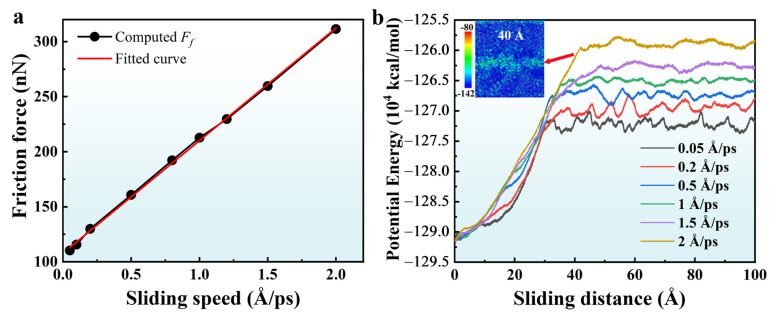
(**a**) Average friction force friction coefficient as a function of sliding speed and (**b**) potential energy curves with sliding distance under different sliding speeds for a contact pressure of 5 GPa for the A5T32 system.

Figure 8 shows the averaged friction force and potential energy for applied pressures of 5 GPa for the A5T32 system under friction velocities from 0.05 to 2 Å/ps. The averaged friction force is obtained in the same way as in Section 2.1. As shown in Figure 8a, the averaged friction force increases from 110 nN (0.05 Å/ps) to 311 nN (2 Å/ps) and is proportional to the sliding speed. The relationship between the average friction force and sliding speed conforms to the formula,

(2) $$F_{f}=f_{0}+kv$$

where *F_f_* is the average friction force, *v* is the sliding speed, and the values of *f*_0_ and *k* can be obtained from the intercept and slope of the fitting curve in Figure 8a. These values are 107.80 and 102.31, respectively. We also find that the relationship between the friction coefficient and the sliding speed also follows the linear formula, $\mu =\mu_{0}+kv$. The values of *µ*_0_ and *k* are 1.02 and 0.96, respectively, which are obtained from the fitting. Figure 8b shows the potential energy curves with sliding distance under different sliding speeds for contact pressures of 5 GPa for the A5T32 system. It can be seen that the potential energy curves of different sliding speeds have the same characteristics, the potential energy increases with the increase in sliding distance and then reaches the dynamic stability value. This is primarily due to the decreasing proportion of cubic diamond structures and the increasing proportion of the cubic diamond (first/second neighbor) lattice and amorphous structures (as shown in Figure 2b) as the sliding distance increases, leading to structural instability and, consequently, an increase in potential energy. The potential energy distribution at 40 Å with a sliding speed of 2 Å/ps, as shown in Figure 8b, reveals that the potential energy increase primarily occurs in the interface region. The potential energy at the interface increases, leading to an increase in the frictional force during the sliding process, as shown in Figure 6. In Figure 2b, the proportions of various structural types stabilize at 240 ps (60 Å) and 300 ps (75 Å), while the potential energy in Figure 8b reaches a dynamic equilibrium value after 40 Å. The trend of the potential energy curve is consistent with the law of friction force in Figure 6, which indicates that high potential energy requires high friction force. It is worth noting that the final potential energy increases with the increases in the friction velocity. This can explain the increase in friction force with the increase in sliding speed.

### 2.3. Roughness Effects of Contact Interface

**Figure 9:**
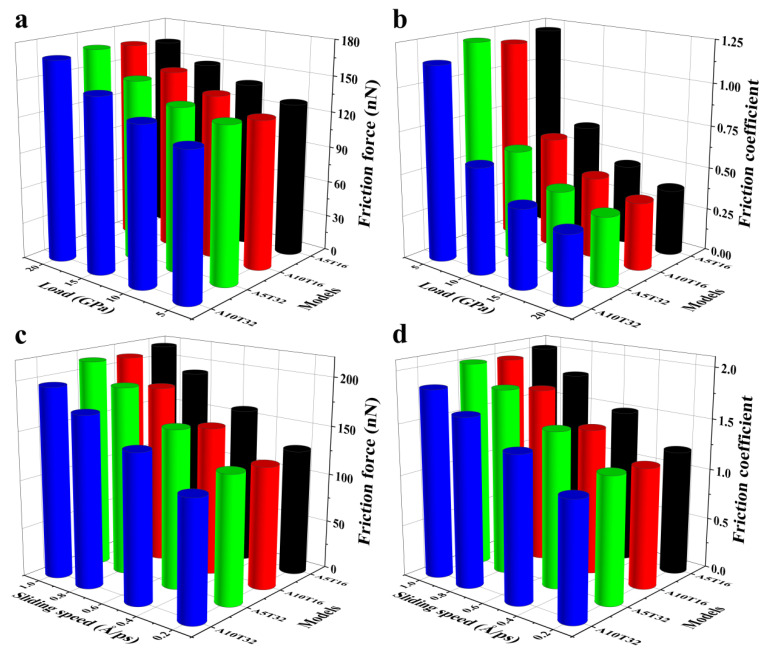
Dependence of (**a**,**c**) average friction force and (**b**,**d**) friction coefficient on the pressures and sliding speeds of different systems.

The frictional properties between the planar upper Si film and lower Si films (A5T16, A10T16, A5T32, and A10T32) with different roughness values at friction velocities of 0.2, 0.5, 0.8, and 1 Å/ps are analyzed, with the normal load set to 5, 10, 15, and 20 GPa, respectively. Figure 9 illustrates the dependence of (a,c) the averaged friction force and (b,d) the friction coefficient on the pressure and sliding speed of different systems. Similar friction behaviors in different systems are shown in Figure 9. That is, the friction coefficient and pressure show an obvious exponential decrease; the average friction force and friction coefficient increase linearly with the sliding speed. This shows that the conclusions drawn in Section 2.1 and Section 2.2 are universal with respect to the planar upper Si film and lower Si films with different roughnesses under dry friction conditions. In Figure 9, it can be seen that the amplitude of the sinusoidal has a larger effect on the frictional properties than the period. For the morphology of the sinusoidal peak with the same period, the averaged friction force and friction coefficient of the system decrease with the increasing amplitude. However, for the morphology of sinusoidal sharp systems with the same amplitude with different periods, the averaged friction force and friction coefficient change very little. The results show that the friction performance of A10T32 is the best among the four rough surfaces, which means that the Si pattern should be etched as deep as possible but not too densely in the preparation of the low-friction-coefficient film. This can effectively improve the operational lifetime of MEMS/NEMS.

## 3. Models and Methods

**Figure 10:**
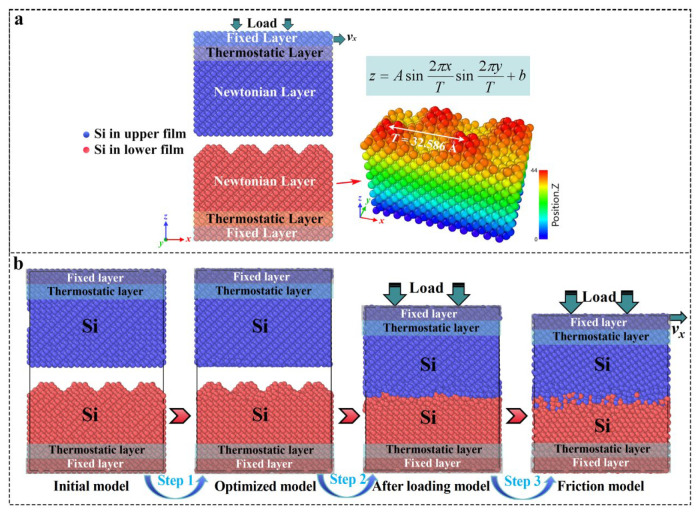
(**a**) Friction model composed of Si film with a sinusoidal surface as the lower layers and a smooth Si film as the upper mating layer. (**b**) Molecular dynamics simulation process diagram.

All molecular dynamics (MD) calculations were performed using the Large-scale Atomic/Molecular Massive Parallel Simulator (LAMMPS) software package (29 August 2024) [34]. The friction model was composed of a planar upper monocrystalline Si film with a size of 65.17 × 32.59 × 48.88 Å^3^ and 5184 Si atoms and a lower Si film with a textured surface, as shown in Figure 10a. The lower, textured Si film was obtained by tailoring the initial monocrystalline Si film; each textured structure had a sinusoidal shape ($z=A\sin\frac{2\pi x}{T}\sin\frac{2\pi y}{T}+b$), in which the amplitude *A* in the *z* direction ranged from 5 to 10 Å, and the period *T* in the *x* and *y* directions changed from 16.29 to 32.59 Å. Based on the different textured amplitude and period values, the corresponding textured Si films were named A5T16, A10T16, A5T32, and A10T32. The MD time step was set as 0.25 fs. Because of the limitations of the MD simulation, the textured parameters were limited to the nanoscale, which was considerably smaller than the textured parameters in the experiment. The post-processing of the dump files was carried out in OVITO (version 3.8.5) [35]. In addition, these nanoscale sizes have been proven sufficient for extracting the crucial information of the friction interface in situ by Li et al. [32]. So, this work also can provide design guidance for the Si film—specifically, those with nanometer thickness for applications in micro/nano-electromechanical system (MEMS/NEMS) devices.

The ReaxFF potential was employed to describe interatomic interactions. It is capable of accurately simulating bond breaking, bond formation, and charge transfer through its use of a bond-order formalism and polarizable charge description. The interatomic forces in the ReaxFF method were obtained from a general energy expression,

(3) $$E_{system}=E_{bond}+E_{over}+E_{under}+E_{lp}+E_{val}+E_{vdWaals}+E_{Coulomb}$$

where the partial contributions to the total energy are the bond, over-coordination penalty and under-coordination stability, lone pairs and valence angle, and non-bonded van der Waals and Coulombic energies, respectively. ReaxFF parameters are optimized against a training set comprising both quantum chemical calculations and experimental data. In the current work, the ReaxFF parameters developed by Narayanan et al. were used for the Si interatomic interactions [36]. The training process for the force field parameters and its comparison with first-principles calculations are described in Refs. [36,37,38]. These works reported good agreement between the ReaxFF results and ab initio data for a variety of systems, such as small clusters, condensed systems, reactive silicon oxides, and solid and liquid Al/α-Al_2_O_3_ interfaces, among others. In addition, ReaxFF has been successfully adopted to simulate the friction process of the silicon interface [39,40]. This validated its accuracy for our systems.

The upper and lower Si films in Figure 10a were divided into three parts: a fixed layer, a thermostatic layer, and a Newtonian layer. The fixed layer ensured the structural stability of the system, the thermostatic layer was kept at 300 K with a Langevin thermostat, while the Newtonian layer was used to simulate the friction-induced structural evolution at the interface. Periodic boundary conditions were applied in both the *x* and *y* directions to eliminate limitations imposed by the model size, and shrink-wrapping was employed along the *z* direction. The simulation was divided into three stages, as depicted in Figure 10b. The first stage was system relaxation: the systems were kept at 300 K for 10 ps with the canonical ensemble (NVT) to achieve an equilibrium state. The second stage was load: the normal load was applied to the fixed layer of the upper Si film until the specified contact pressure value was reached for 10 ps. The third stage was the friction shearing processes: the load was held while the fixed layer of the upper Si film was given a constant sliding speed along the *x* direction to slide for 100 Å (corresponding to slide for 500 ps). Table 1 provides different contact pressures and sliding speeds of the different friction systems.

## 4. Conclusions

The tribological properties of the interaction between a planar upper Si film and lower Si films with different roughnesses under dry friction conditions were determined under different normal loads and sliding speeds using molecular dynamics simulations. The main conclusions are as follows:

(1) The friction force increases with an increase in the normal load under the same sliding speed of 0.2 Å/ps. In the range of 4 GPa–8 GPa, the friction force deviates from the linear relationship. The approximate steady value of the friction coefficient is 0.39, which is in good agreement with the experimental result of 0.37. The shear deformation occurs at the upper Si films, approximately 10 Å above the interface, at a height of z ≈ 44.07 Å.

(2) Under the same normal load (5 GPa), the friction force increases linearly from 110 nN (0.05 Å/ps) to 311 nN (2 Å/ps). The increase in the friction force and friction coefficient is attributed to the increase in potential energy at the interface with the increase in sliding speed.

(3) The frictional properties of the morphology of the sinusoidal surface depend more on the roughness amplitude than the period. The friction performance of A10T32 is the best among the four rough surfaces, which means that the Si pattern should be etched as deep as possible but not too densely in the preparation of the low-friction-coefficient film. This provides theoretical support for the further design of long-operational-lifetime MEMS/NEMS devices.

## Figures and Tables

**Table 1:** Contact pressures and sliding speeds for different models.

Models	Load/GPa										*V_x_*/Åps^−1^								
A5T32	5										0.05	0.1	0.2	0.5	0.8	1	1.2	1.5	2
	2	3	4	5	6	7	8	10	15	20	0.2								
A10T32	5										0.2		0.5		0.8		1		
	10				15				20		0.2								
A5T16	5										0.2		0.5		0.8		1		
	10				15				20		0.2								
A10T16	5										0.2		0.5		0.8		1		
	10				15				20		0.2								

## Data Availability

The original contributions presented in this study are included in the article. Further inquiries can be directed to the corresponding authors.
